# Chain-Breaking Antioxidant and Peroxyl Radical Trapping Activity of Phenol-Coated Magnetic Iron Oxide Nanoparticles

**DOI:** 10.3390/antiox11061163

**Published:** 2022-06-14

**Authors:** Stefano Scurti, Daniele Caretti, Fabio Mollica, Erika Di Antonio, Riccardo Amorati

**Affiliations:** 1 Department of Industrial Chemistry “Toso Montanari”, University of Bologna, UdR INSTM of Bologna, Viale Risorgimento 4, 40136 Bologna, Italy; stefano.scurti2@unibo.it (S.S.); daniele.caretti@unibo.it (D.C.); 2 Department of Chemistry “G. Ciamician”, University of Bologna, Via San Giacomo 11, 40126 Bologna, Italy; fabio.mollica3@unibo.it (F.M.); erika.diantonio@studio.unibo.it (E.D.A.)

**Keywords:** iron oxide, NDGA, SPION, kinetics, autoxidation, peroxidation, magnetic, nanoantioxidant, nanoparticle, ROS

## Abstract

Superparamagnetic iron oxide nanoparticles (SPION) are important materials for biomedical applications, and phenol capping is a common procedure to passivate their surface. As phenol capped SPION have been reported to behave as antioxidants, herein, we investigate the mechanism underlying this activity by studying the reaction with alkyl peroxyl (ROO^•^) radicals. SPION were prepared by coprecipitation of Fe(II) and Fe(III), using phenolic antioxidants (gallic acid, Trolox and nordihydroguaiaretic acid) as post-synthesis capping agents and by different purification procedures. The reactivity of ROO^•^ was investigated by inhibited autoxidation studies, using styrene as an oxidizable substrate (solvent MeCN, 30 °C) and azo-bis(isobutyronitrile) as a radical initiator. While unprotected, bare SPION behaved as prooxidant, accelerating the *O*_2_ consumption of styrene autoxidation, phenol capping provided a variable antioxidant effect that was dependent upon the purification degree of the material. Thoroughly washed SPION, containing from 7% to 14% (*w*/*w*) of phenols, had a low reactivity toward peroxyl radicals, while SPION with a higher phenol content (46% to 55%) showed a strong radical trapping activity. Our results indicate that the antioxidant activity of phenol-capped SPION can be caused by its release in a solution of weakly bound phenols, and that purification plays a major role in determining the properties of these materials.

## 1. Introduction

Superparamagnetic iron oxide nanoparticles (SPION) are an interesting class of nanomaterials that, due to their possibility to interact with external magnetic fields, are employed in a number of biomedical applications, including hyperthermia for cancer treatment; magnetic resonance imaging; the targeted delivery of drugs, antibodies, and nucleic acids; biosensing; and the separation of biomolecules [1,2]. Furthermore, the presence of redox active iron ions makes SPION potentially suitable for counteracting cancer by the induction of ferroptosis [3], as well as for environmental bioremediation [4], due to the production of free radicals via Fenton chemistry [5]. Surface modification plays a fundamental role in determining the effectiveness of such materials, and this is typically performed by employing inert inorganic layers (gold, silica) or biocompatible materials, including polysaccharides, poly(ethylene glycol), poly(vinyl pyrrolidone), or small molecules, such as fatty acids and amino acids [2]. Phenols and polyphenols have long been used as surface modifiers of SPION, due to their affinity to iron ions, positive influence on the overall magnetization of the nanoparticle, and the possibility of using them as a reductant during the synthesis [6]. Examples reported in the literature are mainly represented by bidentate catechol derivatives comprising gallic acid [7], gallotannins [8], dopamine [9], caffeic acid [10], quercetin [11] and monophenols with acid functionalities that can help to bind the metal surface, such as curcumin [12] and 2-hydroxyisophthalic acid [13]. Because of the widespread presence of polyphenols in the vegetal kingdom, a number of protocols employing plant extracts functioning at the same time as reductant and surface capping agents have been proposed [14,15]. Given the well-known ability of phenols to react with free radicals by H-atom or electron donation [16], it is not surprising that iron oxide nanoparticles with phenolic surface capping have been proposed to possess antioxidant activity, based mainly on the ability to quench stable radicals such as 2,2-diphenyl-1-picrylhydrazyl (DPPH^•^) [17,18,19,20]. However, because of the known shortcomings of assays based on stable radicals [21], further studies are necessary to fully clarify the mechanism underlying this potentially important property and the role of phenol capping. In fact, because of the absence of an oxidizable substrate, the DPPH^•^ assay cannot predict if SPION-derived materials are pro-oxidant or able to trigger the peroxidation of lipids. Moreover, stable radicals are chemically different from the reactive oxygen species (ROS) present in real systems, and thus the assays based on them may be prone to either false-positive or -negative results that should be confirmed by methods based on inhibited autoxidation [21]. Herein, we report for the first time the ability of SPION, capped with different phenols, to trap alkylperoxyl radicals (ROO^•^), which are the ROS responsible for the peroxidation of polyunsaturated fatty acids and the autoxidation of organic materials in general [22]. Herein, this inhibition of the oxidative radical chain is defined as “chain-breaking” antioxidant effect [23]. The relevance of this study is supported by the importance of reducing the radical-generating tendency of iron nanoparticles for biomedical purposes [23,24] and obtaining “magnetic” antioxidants that are able to be directed or removed simply by using an external magnetic field [25,26,27].

## 2. Materials and Methods

### 2.1. Materials

Iron(III) chloride (FeCl_3_), ammonium iron(II) sulfate hexahydrate ((NH_4_)_2_Fe(SO_4_)_2_(H_2_O)_6_), 3,4,5-trihydroxybenzoic acid (gallic acid), 6-hydroxy-2,5,7,8-tetramethylchroman-2-carboxylic acid (Trolox), nordihydroguaiaretic acid (IUPAC name: 4-[4-(3,4-dihydroxyphenyl)-2,3-dimethylbutyl]benzene-1,2-diol), sodium hydroxide (NaOH), styrene, 2,2′-azobis(2-methylpropionitrile) (AIBN), 2,2,5,7,8-pentamethyl-6-chromanol, *tert*-butylhydroperoxide, di-*tert*-butylperoxide, and hydrogen peroxide (50% *w*/*w*) were purchased from Sigma Aldrich and directly used unless otherwise specified. Styrene was purified from the inhibitor by percolating it on alumina/silica column, AIBN was recrystallized from methanol. The solvents were HPLC grade and were used without further processes of purification.

### 2.2. Synthesis and Purification of SPION

Co-precipitation synthesis was used to prepare the super paramagnetic iron oxide nanoparticles (SPION), employing FeCl_3_ and (NH_4_)_2_Fe(SO_4_)_2_·6H_2_O (Mohr’s salt) as nanoparticles precursors. The reaction was carried out in a three-neck flask under nitrogen atmosphere by FeCl_3_ (12.7 mmol) and Mohr’s salt (6.4 mmol) in a Fe^3+^: Fe^2+^ molar ratio of 2: 1 in 100 mL of water. To promote the formation of mixed oxide, sodium hydroxide was added as a base. The solution was stirred for 30 min at 70 °C, and the obtained nanoparticles were filtered using a Buchner funnel and washed several times with distilled water to remove ionic species. The synthesized nanomaterials were characterized by infrared spectroscopy (IR), thermogravimetric analysis (TGA), X-ray diffraction (XRD) and scanning electron microscopy (SEM), and the results obtained were in agreement with those reported in our recent work [28].

Phenol-coated magnetite nanoparticles were prepared by surface functionalization approach [28]. The as-synthetized SPION (1 g) were suspended in 10 mL of a freshly prepared water concentrated solution containing the ligand (10% *w*/*w*). The mixture was vigorously stirred for 24 h at room temperature by a magnetic stirrer. The obtained coated SPION were filtered on filter paper using a Buchner funnel and washed three times with acetone directly poured on the filter under vacuum, to remove the excess of the ligands. This procedure provided a pseudo core–shell system containing approximatively 50% *w*/*w* of organic coating (see below).

These samples were then washed with MeCN several times into a quartz cuvette, and the surnatant was analysed by UV–vis spectroscopy until it did not show the typical absorption spectrum of phenols. The obtained samples were thus dried under high vacuum overnight.

### 2.3. Characterization of SPION

The bare and coated SPION were characterized by Fourier-transform infrared spectroscopy (FTIR), which acquired the spectra through an ATR-IR Bruker Alpha I spectrometer. To evaluate the amount of organic coating and thermal stability, TGA analyses were carried out using a thermogravimetric apparatus (TA Instruments Q500 New Castle, DE, USA) under a nitrogen atmosphere (flow rate 60 mL/min) at 20 °C/min heating rate, from 20 to 450 °C. TGA sensitivity was 0.1 μg with a weighting precision of ±0.01%. Powder XRD patterns were recorded on a PANalytical X’PertPRO X-ray diffractometer using a Cu radiation source (λ = 1.54 Å). Diffraction patterns were recorded between 10° and 80° 2θ over a scan time of 10 min. SPION were also characterized by a scanning electron microscope Thermo Phenom Prox (SEM) to evaluate size and morphology. Finally, dynamic light scattering (DLS) analyses were carried out by a Malvern Zetasizer Nano ZS instrument. The experimental temperature was set as 25 °C. Moreover, the poly dispersity index (PDI) was evaluated based on a scale from 0 to 1, where the value of 0 represented the mono-dispersity condition.

### 2.4. Inhibited Autoxidation Studies

Autoxidation experiments were performed by measuring the oxygen consumption in a two-channel gas uptake apparatus, immersed in a thermostatic bath and based on Validyne DP15 pressure transducer. The rate of initiation (*R_i_*) was calculated in preliminary set of experiments from the length of the inhibition period, τ, using 2,2,5,7,8-pentamethyl-6-chromanol as a reference antioxidant during autoxidation of styrene [29]. The results are the mean of at least two repeated experiments.

## 3. Results

### 3.1. Synthesis and Characterization

**Scheme 1:**
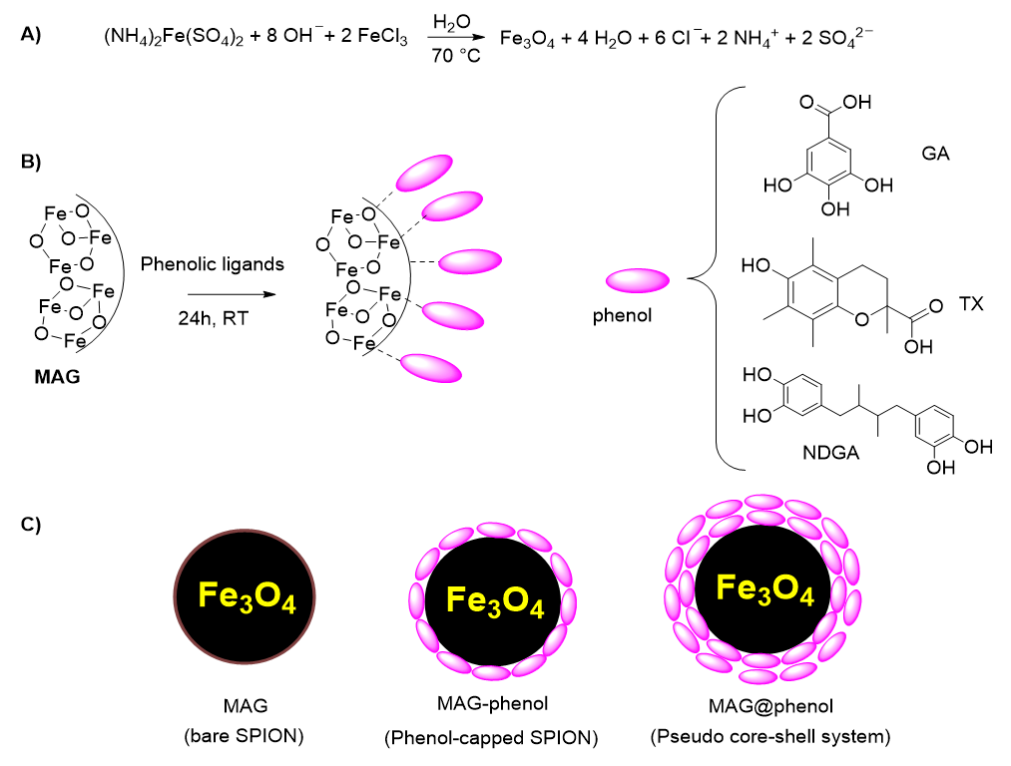
Schematic representation of the synthetic route employed to prepared phenol−coated SPION and the names of the samples investigated: (**A**) synthesis of the magnetic core; (**B**) phenol coating; (**C**) different samples obtained after washing with solvents.

With the aim of preparing nano-antioxidant materials with magnetic properties, Fe_3_O_4_ nanoparticles were synthetized by the co-precipitation method using a Fe^3+^: Fe^2+^ molar ratio of 2:1 and NaOH. Bare SPION (herein, MAG) presented a hydrodynamic diameter of 46 nm by DLS determination and were then functionalized with phenolic ligands to obtain MAG@phenol samples (Scheme 1). A post functionalization approach was used to avoid degradative phenomena that may affect phenols at the relatively high temperature of the synthesis. Phenols were chosen to cover different class of antioxidants presenting in nature and typically employed in SPIONs synthesis. Gallic acid (GA) is a versatile antioxidant with anticancer, anti-melanogenic and anti-steatosis applications [30,31] and was previously used as capping agent for SPION [7]. Nordihydroguaiaretic acid (NDGA) is a natural phenolic lignan known for its lipoxygenase-inhibiting properties [32] and is actively investigated for its promising activity against chronic diseases [33]. Trolox (TX) is an artificial α-tocopherol analogue that is popular as a water-soluble reference in antioxidant tests [34].

**Figure 1:**
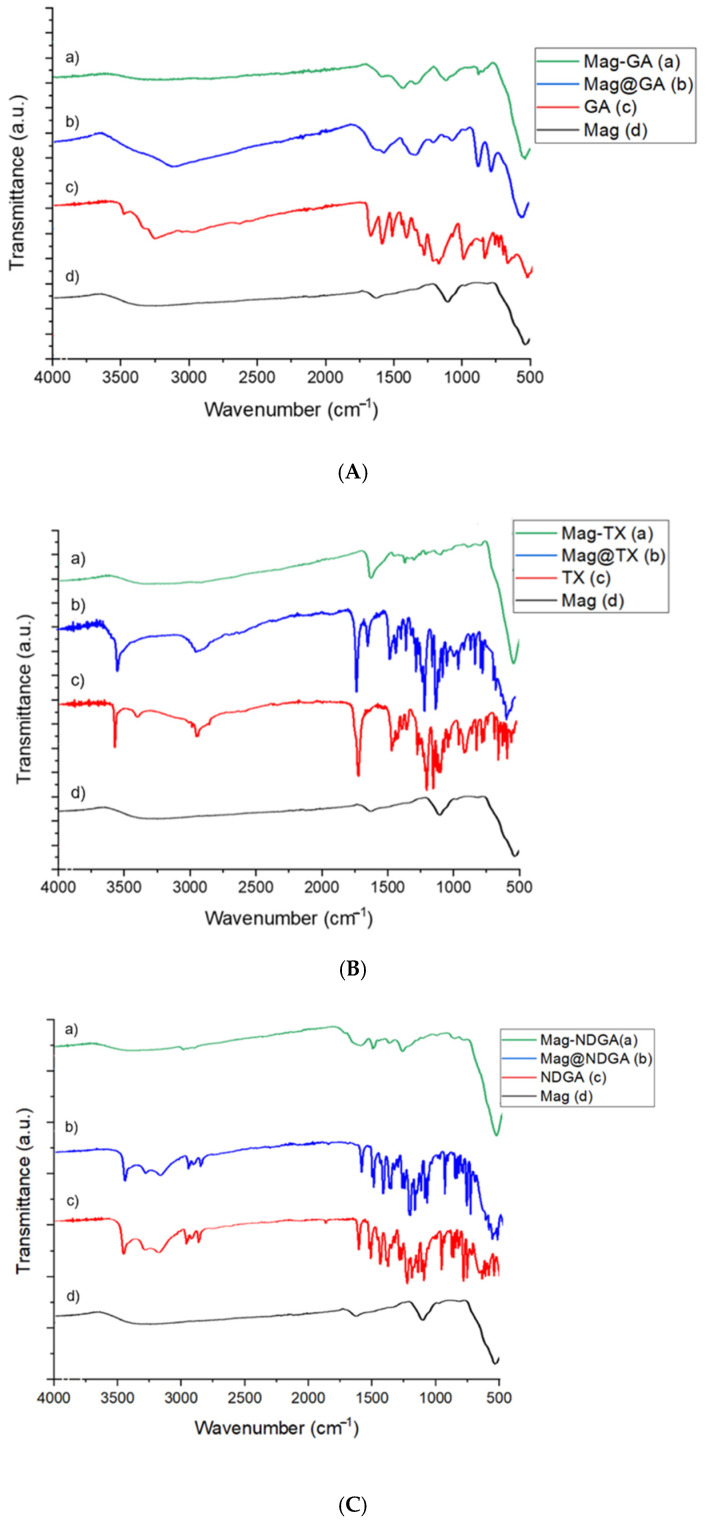
Panels (**A**–**C**): FT-IR spectra of the investigated materials obtained from gallic acid (**A**), Trolox (**B**) and nordihydroguaiaretic acid (**C**).

Functionalization was confirmed by FT-IR spectroscopy, as shown in Figure 1, in which the IR spectra related to the particles coated with gallic acid (GA), Trolox (TX) and nordihydroguaiaretic acid (NDGA) are displayed and compared with the uncoated Fe_3_O_4_ nanoparticles. The peaks of 580 cm^−1^ and 1100 cm^−1^ in bare magnetite nanoparticles were assigned to Fe-O and Fe-OH bond stretching motion, respectively [28]. When comparing MAG with the functionalized system, a series of signals related to the organic coating can be observed. However, the Fe-O stretch was present in all samples, suggesting the correct functionalization of SPIONs.

**Figure 2:**
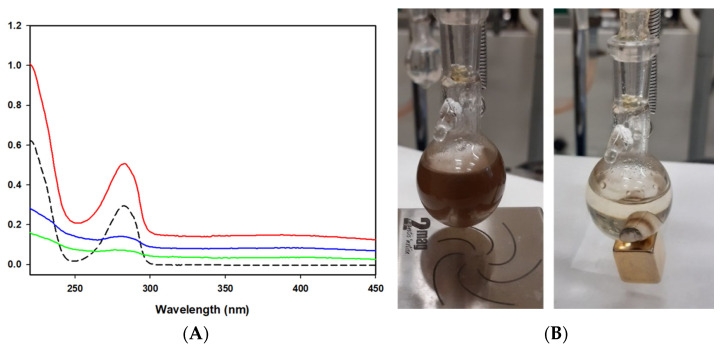
(**A**) UV–vis spectra of the surnatant during the washing procedure of MAG@NDGA showing the decrease in absorption due to the released NDGA after the washing cycles (1° = out of scale, not shown, 2° = red, 3° = blue, 4° = green), dashed line represents reference NDGA (0.04 mM); (**B**) dispersion of SPION under stirring with a magnetic stir bar (left) and precipitation by applying a neodymium magnet (right).

The MAG@phenol samples were then subjected to repeated washing cycles with MeCN into a quartz cuvette. The washing procedure was carried on until the surnatant did not show the typical absorption spectrum of phenols at UV–vis spectroscopy (see for example MAG@NDGA in Figure 2A). The nanomaterials obtained are therefore assumed to consist of phenol-capped SPION, herein named as MAG-phenol (see Scheme 1).

**Figure 3:**
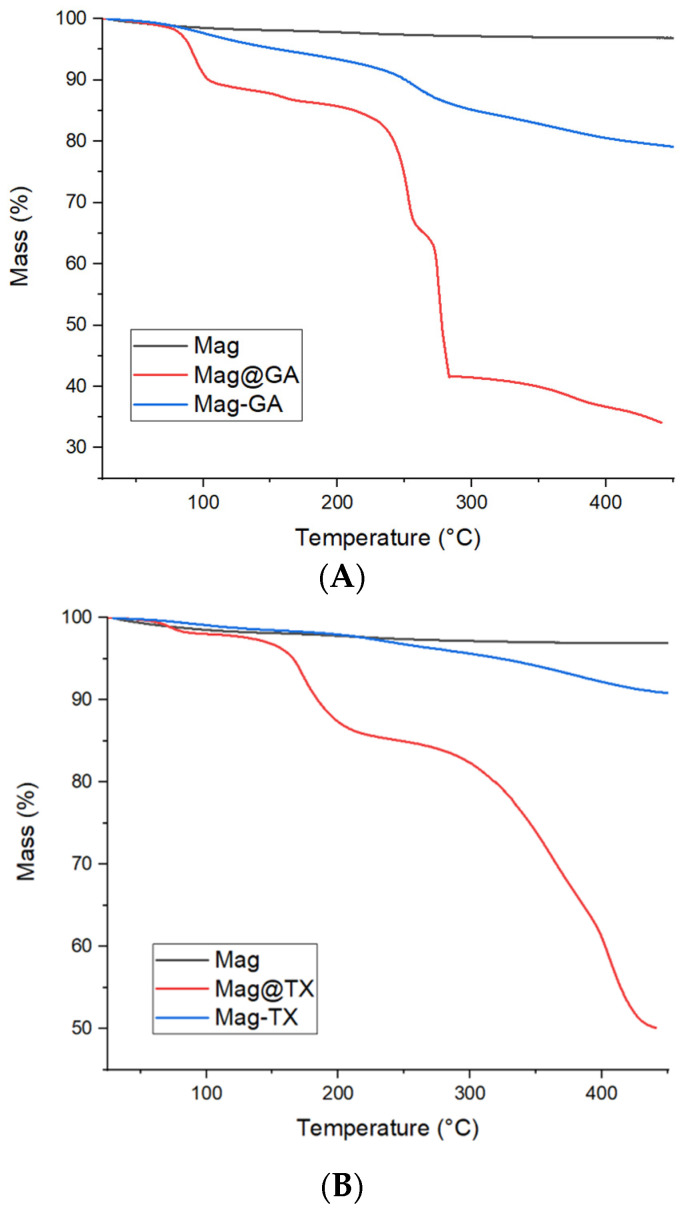

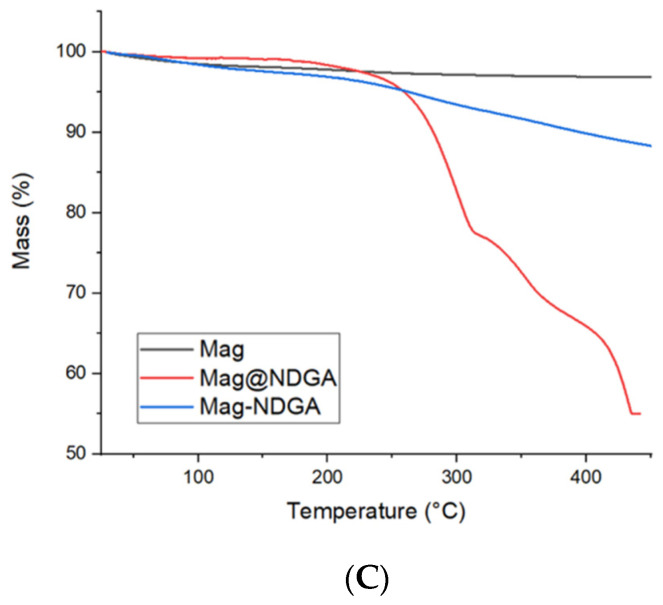
Panels (**A**–**C**): TGA thermograms of the investigated materials obtained from gallic acid (**A**), Trolox (**B**) and nordihydroguaiaretic acid (**C**).

A thermo-gravimetric analysis was then carried out to evaluate the effect of acetonitrile washing on the amount of coating. As Figure 3 shows, the phenol-coated SPION displayed a weight loss indicating the presence of an organic component that was absent in bare SPION. Weight loss between 100 and 450 °C was used to measure the phenol loading. In agreement with the UV–vis spectra recorded during the washing procedure, the phenols presented on the SPIONs surface decrease during the washing phase.

In this way, the system switched from a multilayer product where the phenols in the nanoparticles were coordinated between them to form a sort of phenol shell (pseudo core–shell system) with a pseudo-mono layer of phenol coating. The percentage of organic coating before and after the washing is shown in Table 1.

The reduction in phenol coating was also confirmed by the IR spectra, which showed that the signals related to the organic coating decrease in intensity (Figure 1) after washing with acetonitrile. The multi-layer phenol capping in case of MAG@phenols was ascribed to phenol precipitation during the synthesis.

### 3.2. Antioxidant Activity

**Scheme 2:**
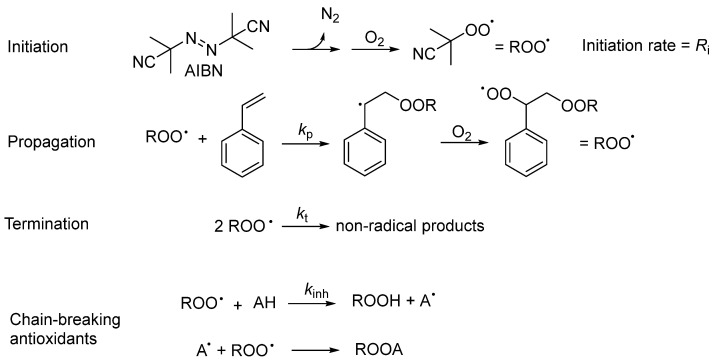
Reaction scheme of styrene autoxidation inhibited by a chain-breaking antioxidant *AH*. *R* = alkyl chain.

The antioxidant activity was studied using the inhibited autoxidation method, that consists of measuring the *O*_2_ consumption during the autoxidation rate of an organic substrate (in our case, styrene; see Scheme 2) in the absence or in the presence of a chain-breaking antioxidant *AH* [35]. The reaction is initiated by the thermal decomposition of 2,2′-azobis(2-methylpropionitrile) (AIBN) at 30 °C, as shown in the “initiation” step in Scheme 2. After AIBN fragmentation, alkylperoxyl radicals (ROO^•^) are generated at a constant rate *R_i_* (rate of initiation). Then, ROO^•^ radicals react with styrene (with a rate constant of propagation *k_p_*), forming alkyl radicals (R^•^) that react at a diffusion-controlled rate with *O*_2_, finally yielding new ROO^•^ radicals. This propagation step is repeated many times until the ROO^•^ radicals disappear by bimolecular recombination, with rate constant *k*_t_ (Scheme 2, termination step). In the presence of a chain-breaking antioxidant *AH*, ROO^•^ radicals are first trapped by H-atom transfer (rate constant *k_inh_*) and then by a fast radical-radical reaction between the A^•^ and ROO^•^, that may consist of a recombination (as shown in Scheme 2) in the case of monophenols such as Trolox or H-atom transfer in the case of catechols [35,36]. Because of these reactions, phenolic antioxidants usually show a stoichiometry of radical trapping (*n*) equal to 2.

For a kinetic description of the inhibition by antioxidants, Equations (1) and (2) can be derived by applying the steady state approximation to R^•^ and ROO^•^ species, and by assuming that all ROO^•^ disappear by reacting with *AH* (termination by chain-breaking antioxidants in Scheme 2) [35]. This mathematical analysis was shown to correctly describe the behaviour of antioxidants at low temperatures (i.e., if ROOH decomposition is negligible) with different oxidizable substrates (methyl linoleate [37], tetrahydrofuran [38], squalene [39], etc.) under various conditions (homogeneous solutions [37,38,39], micelles [40], liposomes [41]). Equation (1) indicates that the duration of the inhibition period (τ) is proportional to *AH* concentration and to the stoichiometry of the reaction of *AH* with radicals, *n*, while is inversely proportional to the initiation rate *R_i_*. The rate constant of the reaction of ROO^•^ with *AH* (*k_inh_*) is obtained using Equation (2), in which *k_p_* is the propagation rate constant of styrene (41 M^−1^s^−1^ at 30 °C) [37] and −(*d*[*O*_2_]/*dt*)_inh_ is the slope of *O*_2_ consumption during the inhibition period [35]:

(1) $$R_{i}=\frac{n\left[AH\right]}{\tau }$$

(2) $$-{\left(\frac{d\left[O_{2}\right]}{dt}\right)}_{inh}=\frac{k_{p}\left[styrene\right]R_{i}}{nk_{inh}\left[AH\right]}$$

**Figure 4:**
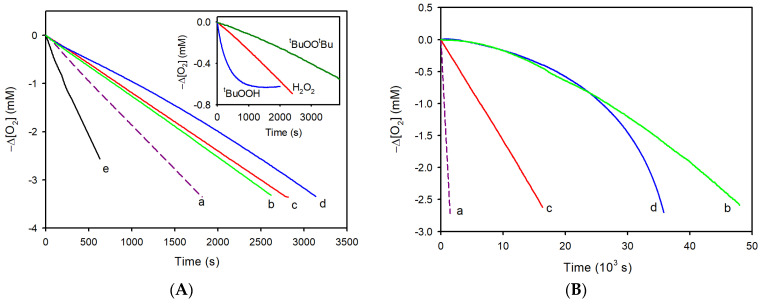
Oxygen consumption measured during the autoxidation of styrene (4.3 M) in MeCN, initiated by AIBN (25 mM) at 30 °C, in the absence of inhibitors (a, dashed line) or in the presence of the investigated antioxidants: Panel (**A**): MAG-NDGA (b), MAG-GA (c), MAG-TX (d), bare MAG (e), (all 0.25 mg/mL); Panel (**B**): NDGA (0.082 mM) (b), GA (0.18 mM) (c), TX (0.065 mM) (d), corresponding to the phenol concentration of the experiments reported in Panel (**A**). Inset in Panel (**A**) reports the *O*_2_ consumption during the autoxidation of styrene (4.3 M) in MeCN in the presence of MAG (0.25 mg/mL) and 10 mM of different peroxides.

**Figure 5:**
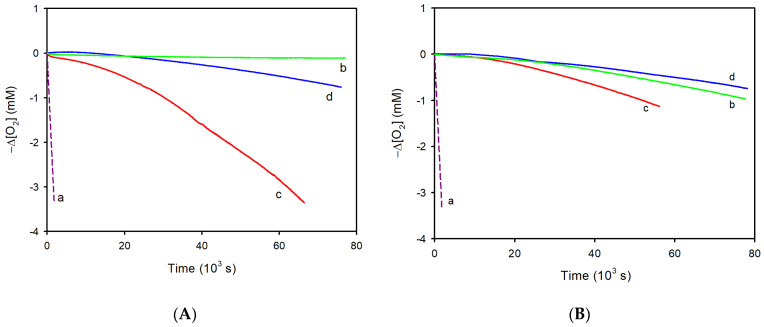
Oxygen consumption measured during the autoxidation of styrene (4.3 M) in MeCN, initiated by AIBN (25 mM) at 30 °C, in the absence of inhibitors (a, dashed line) or in the presence of the investigated antioxidants: Panel (**A**): MAG@NDGA (b), MAG@GA (c), MAG@TX (d), (all 0.25 mg/mL); Panel (**B**): NDGA (0.54 mM) (b), GA (0.83 mM) (c), TX (0.40 mM) (d), corresponding to the phenol concentration of the experiments reported in Panel (**A**).

In the case of antioxidants providing only a retardation of *O*_2_ consumption, a different equation was used as detailed in Appendix A. The stoichiometric coefficient was assumed to be 2 for GA and TX and 4 for NDGA as it is a bifunctional phenol [29]. The SPION were well-dispersed in the reaction medium by stirring with a magnetic stir bar, as shown in Figure 2B, which shows a picture of the SPION during the experiments. SPION could be recovered by using an external neodymium magnet. Bare SPION (MAG) and phenol-capped SPION (MAG@phenols and MAG-phenols) were investigated as inhibitors of the autoxidation of styrene at 30 °C in acetonitrile. The obtained *O*_2_ consumption plots are reported in Figure 4 and Figure 5, while the slopes of *O*_2_ consumption and the calculated *k_inh_* are shown in Table 2.

In the case of phenol-capped SPIONs, a kinetic analysis was performed using the concentration of phenols as obtained by the TGA experiments. By comparing the dashed and the solid lines in Figure 4A, it is evident that bare SPION have a pro-oxidant effect, as they increase the *O*_2_ consumption rate. This aspect was studied in greater detail by performing styrene autoxidation experiments, in which the initiation was not caused by the decomposition of the azoinitiator but was due to the Fenton reaction between MAG and peroxides. In the inset of Figure 4A, the *O*_2_ consumption measured during the styrene autoxidation initiated by SPIONs in the presence of *^t^*BuOOH, H_2_*O*_2_ or *^t^*BuOO*^t^*Bu is reported. The results show that a higher production of radicals is observed for hydroperoxides in the order *^t^*BuOOH > H_2_*O*_2_, followed by the dialkylperoxide *^t^*BuOO*^t^*Bu. While a deeper exploration of these results goes beyond the aims of this work, we show that the dialkylperoxides formed during styrene autoxidation (see Scheme 2) can provide a pro-oxidant effect in the presence of bare SPION. Moreover, SPION capped with phenols and subjected to repeated washing cycles have a weak antioxidant activity as they afford only a retardation of styrene autoxidation (Figure 4A). At the same concentration, the investigated phenols have a much higher inhibiting activity, and except for gallic acid, they provide a clear inhibition period (Figure 4B). The *k_inh_* values calculated from these experiments by using the slopes of *O*_2_ consumption at the beginning of the inhibition period, show that ligation of SPION caused a 10-to-100-fold decrease in *k_inh_* (see Table 2).

Therefore, it can be concluded that binding to the nanoparticle surface reduces the reaction of phenols with ROO^•^, but at the same time, completely suppresses the pro-oxidant behaviour of the MAG core. Phenol-capped SPION, subjected only to washing with acetone, afforded a very strong inhibition of styrene autoxidation for a long time as the effect of the release of weakly bound phenols from the surface of the nanoparticles (Figure 5A). In this case, the antioxidant activity was very similar to that of phenols at the same concentration (Figure 5B), except for MAG@GA, which showed a reduced activity with respect to GA.

## 4. Discussion

### 4.1. Phenol Capping Suppresses Pro-Oxidant Effect

The measure of the autoxidation rate of an organic substrate in the presence and absence of a supposed inhibitor makes it possible to investigate not only the radical trapping ability, but also the pro-oxidant activity that derives from the interaction with the peroxides, which are inevitably produced under the same conditions. While for most phenolic and arylamine antioxidants, pro-oxidant activity is marginal, this is not true for many nanoantioxidants with metal and metal oxide cores, as they might catalyse the homolytic decomposition of peroxides [42].

Our experiments show that “bare” SPIONS are capable of accelerating the AIBN-initiated autoxidation of styrene, as expected from the known ability of materials containing surface Fe^2+^ ions to participate in heterogeneous Fenton chemistry [43]. Considering that styrene autoxidation proceeds via the formation of a polyperoxide (see Scheme 2) [37], this observed acceleration is due to the homolytic dissociation of O-O styryl polyperoxide affording alkoxyl radicals [44]. We also show that the heterogeneous Fenton reaction between bare SPION and three peroxides is clearly detectable and increases in the order *^t^*BuOO*^t^*Bu < H_2_*O*_2_ < *^t^*BuOOH (inset of Figure 4a).

### 4.2. Antioxidant Activity of Phenol-Capped SPION

Pseudo core–shell SPION (MAG@phenol) were repeatedly washed by using MeCN until no absorption from phenols was visible at the UV–vis spectrum to remove the weakly bound antioxidants. With this procedure, phenol-capped nanoparticles (MAG-phenol) were obtained. Inhibited autoxidation experiments show that MAG-phenols have a weak antioxidant activity. This result can be explained by considering that phenols bind to the iron oxide surface mainly by using the phenolic OH groups, which are therefor not available for H-atom donation to ROO^•^. The residual ROO^•^ trapping activity may derive from traces of loosely bound phenols or from the intrinsic reactivity of the surface. The fact that MAG-NDGA has a reactivity similar to that of the other SPION suggests that both catechol groups of NDGA are involved in binding to the metal surface. Interestingly, the phenol-capped SPION do not have the same pro-oxidant effects as bare SPION, most likely because the phenol-bound iron ions are no longer active, in agreement with the known Fenton-suppressing effect of polyphenolic iron chelators [45]. Moreover, it cannot be excluded that, although the heterogeneous Fenton reaction was not entirely suppressed, the alkoxyl radicals, being extremely reactive [46], may react with the capping phenols.

### 4.3. Antioxidant Activity of Pseudo Core-Shell SPION

In the absence of a thorough washing cycle, pseudo core–shell SPION containing variable amounts of loosely bound phenols were obtained. The phenol loading was therefore determined for each synthesis batch by TGA analysis. The experiments show a very strong inhibition of styrene autoxidation by MAG@phenol, which matches that displayed by unbound phenols, with the exception of gallic acid. Therefore, pseudo core–shell SPION behave as a reservoir of phenols that dissolves in solution and inhibits autoxidation, similar to other systems based on antioxidant loading into nano-scaffolds [47,48]. A possible explanation for the low efficacy of MAG-GA is the slow release of GA in solution.

To the best of our knowledge, extensive washing with a good phenol-solubilizing solvent is not a commonly used procedure to prepare phenol-covered SPION. For example, in some recent works [17,18,19], phenol-capped SPIONs were either not washed at all or washed only with deionized water, whose phenol solubilizing ability may be poor.

In the work by Shah et al., the magnetic nanoparticles were washed three times with acetone, that is a solvent expected to solubilize phenols, but the purification extent is not measured [20]. Therefore, it is possible that many claimed antioxidant activities of phenol-capped SPION are simply due to weakly bound phenols, which are not removed during synthesis and released under the conditions of the antioxidant test. In this regard, it should be underlined that the DPPH^•^ test is performed in ethanol or methanol, which are two effective phenol-solubilizing solvents [21].

## 5. Conclusions

In this study, we showed that SPION capped with gallic acid, Trolox or nordihydroguaiaretic acid have different antioxidant activities depending on the preparation procedure. Extensive washing with an effective phenol-solubilizing solvent provides phenol-capped SPION with a much lower antioxidant activity than the phenols considered alone at the same concentration. This demonstrates that binding to the iron oxide surface hampers the ability of phenols to donate a H-atom to peroxyl radicals. At the same time, this observation suggests that metal–phenol binding could increase the stability of capping phenols in an oxidizing environment, thus also providing a stable capping in biological systems, as demonstrated in various studies [7,12]. Most importantly, phenol-capping avoids the prooxidant effect, which is instead observed with bare SPION.

In addition, by modulating the washing procedure, pseudo core–shell systems are obtained that contain a significant amount of loosely bound phenols, which are released in solution and provide a strong antioxidant effect. Pseudo core–shell SPION represent an interesting platform to obtain phenol release systems, which may be useful in a variety of applications where the magnetic core provides an added value. These results are valuable for understanding the antioxidant activity of iron-oxide-based nanomaterials, especially for biomedical and food purposes.

## Figures and Tables

**Table 1:** Mass percentage of organic coating before and after the washing phase with acetonitrile.

	MAG@phenol ^a^ (%)	MAG@phenol ^b^ (%)
GA	55	14
TX	48	7
NDGA	46	8

^a^ pseudo core–shell system; ^b^ phenol-capped system, after extensive washing with MeCN.

**Table 2:** Rates of *O*_2_ consumption during AIBN-initiated styrene autoxidation and rate constants for the reaction with peroxyl radicals (ROO^•^).

Antioxidant	−*d*[*O*_2_]/*dt*/μMs^−1^	*k_inh_*/M^−1^s^−1^
-	1.8 ± 0.1	-
MAG	4.3 ± 0.3	-
GA	0.16 ± 0.02 ^a^	(1.3 ± 0.3) × 10^4^
TX	0.012 ± 0.002 ^a^	(4.5 ± 0.8) × 10^5^
NDGA	0.05 ± 0.01 ^a^	(8 ± 2) × 10^4^
MAG-GA	1.3 ± 0.2 ^a^	(1.4 ± 0.2) × 10^3^
MAG-TX	1.1 ± 0.2 ^a^	(5.0 ± 0.6) × 10^3^
MAG-NDGA	1.3 ± 0.2 ^a^	(1.3 ± 0.2) × 10^3^

^a^*O*_2_ consumption rates for the autoxidation reported in Figure 4, measured at the beginning of the inhibited period.

## Data Availability

Data are contained within the article.

## Appendix A

In the case of antioxidants that are able to provide only a retardation of the *O*_2_ consumption without a recognizable induction period (see, for instance, traces b, c and d in Figure 4A), Equation (2) is no longer valid because the fraction of ROO• radicals disappearing by self-termination is not negligible. In this case, more reliable *k_inh_* values can be obtained by Equation (A1), which is derived from the same kinetic scheme reported in Scheme 2 but without the assumption that all radicals are terminated by the reaction with the antioxidant:

(A1) $$\frac{{\left(-d\left[O_{2}\right]/dt\right)}_{0}}{{\left(-d\left[O_{2}\right]/dt\right)}_{inh}}-\frac{{\left(-d\left[O_{2}\right]/dt\right)}_{inh}}{{\left(-d\left[O_{2}\right]/dt\right)}_{0}}=\frac{nk_{inh}\left[AH\right]}{\sqrt{2k_{t}R_{i}}}$$

In Equation (A1), −(*d*[*O*_2_]/*dt*)_0_ and −(*d*[*O*_2_]/*dt*)_inh_ are the slopes of *O*_2_ consumption in the absence and presence of the antioxidant, respectively, and *k*_t_ is the termination constant of the autoxidation of styrene (*k*_t_ = 2.1 × 10^7^ M^−1^s^−1^) [49].
